# Protein as Chemical Cue: Non-Nutritional Growth Enhancement by Exogenous Protein in *Pseudomonas putida* KT2440

**DOI:** 10.1371/journal.pone.0103730

**Published:** 2014-08-12

**Authors:** Hiren Joshi, Rachna Dave, Vayalam P. Venugopalan

**Affiliations:** Biofouling and Biofilm Processes Section, Water and Steam Chemistry Division, Bhabha Atomic Research Centre, Kalpakkam, India; University of Padova, Medical School, Italy

## Abstract

Research pertaining to microbe-microbe and microbe-plant interactions has been largely limited to small molecules like quorum sensing chemicals. However, a few recent reports have indicated the role of complex molecules like proteins and polysaccharides in microbial communication. Here we demonstrate that exogenous proteins present in culture media can considerably accelerate the growth of *Pseudomonas putida* KT2440, even when such proteins are not internalized by the cells. The growth enhancement is observed when the exogenous protein is not used as a source of carbon or nitrogen. The data show non-specific nature of the protein inducing growth; growth enhancement was observed irrespective of the protein type. It is shown that growth enhancement is mediated via increased siderophore secretion in response to the exogenous protein, leading to better iron uptake. We highlight the ecological significance of the observation and hypothesize that exogenous proteins serve as chemical cues in the case of *P.putida* and are perceived as indicator of the presence of competitors in the environment. It is argued that enhanced siderophore secretion in response to exogenous protein helps *P.putida* establish numerical superiority over competitors by way of enhanced iron assimilation and quicker utilization of aromatic substrates.

## Introduction

Extracellular secretion of proteins is an evolutionarily conserved phenomenon occurring in all domains of life [1]. These secretory proteins play a critical role in the survival of organisms in the environment by performing an array of “remote control” functions like nutrient uptake, competitor killing, virulence and pathogenicity [1]–[3]. In addition, there are reports indicating dual role of proteins which function in the cytoplasm as well as in the extracellular environment [4]. These observations substantiate the key role played by secretory proteins in microbial persistence in a given ecological niche. Moreover, recent evidence suggests that microbes and host significantly influence the composition of each other's secretory proteins to benefit themselves [5]–[8]. On a similar note, there have been many reports which have indicated involvement of protein in communication among microbes as well as between plants and microbes [5], [9], [10]. For example, Han et al. [9] have reported small protein molecule mediated signaling in *Xanthomonas* sp., while Badri et al. [10] and De-la-Peña et al. [5] have shown the role of protein in plant-plant and plant-microbe interactions, respectively. These observations have clearly established the role of proteins as signaling molecules, a fact that was overlooked for quite long time. Collectively, all these observations along with recent development in soil proteome analysis techniques have clearly indicated the cardinal role played by extracellular proteins in development of community structure, which also signifies their widespread presence in the natural environment.

Many natural habitats are densely populated with microbes having cell density as high as 10^9^–10^11^cell/cm^3^ of sediments [11]. This high density raises the possibility of non-target organisms coming into contact with these extracellular proteins. Most secretory proteins act as nitrogen or carbon source and therefore will have impact over non-target organims in terms of their nutritional requirement. However, many organisms do not have capability to secrete extracellular proteases, a fact that does not alow them to utilize these exogenous proteins. Consequently, it would be quite intresting to know how these protease-negative organims react to the extracellular prtoeins and in what way their reaction would help in their survival in the natural environment.

It has been clearly established that proteins infleunce physiology of many organisms, independent of their nutritional value [5]–[8]. Consequently, one can expect that a non–target, extracellular protease-negative organism may also percieve them and mount certain type of response. On a similar note, signaling molecules are known to influence non-target organisms, mainly due to the molecular similarity between various signaling molecules, leading to the phenomenon termed as cross talk [12], [13]. This essentially facilitates inter-species as well as inter-kingdom communications [14]. These arguments incited us to investigate the non-nutritional influence of extracellular proteins on microbes. The omnipresence of proteins in nature would ensure that many organisms come in close contact with these proteins, eliciting responses that may impact fitness of the organisms concerned. We have carried out experiments on the influence of allochthonous proteins on the physiology of extracellular protease-negative model organism *Pseudomonas putida* KT2440.

Organisms belonging to the genus *Pseudomonas* are among the predominant inhabitants in many ecological niches. They are mostly found in soil rhizospheric environment and in association with host organism like humans. It is well documented that *Pseudomonas* closely interacts with their host organism as well as with plants in the rhizospheric environment [15], [16]. Interestingly, many of these interactions have been shown to be governed by secretory proteins. For example, Kruczek et al. [17] and Kim et al. [18] have shown that serum albumin induces the expression of iron regulated genes and type III secretion in *P.aeruginosa*. On a similar note, De-la-Pena et al. [7] have shown growth inhibitor effect of root secretory protein on *Pseudomonas*. These observations clearly indicate the important role played by extracellular proteins on physiology of *Pseudomonas*. Therefore, we chose the model organism *Pseudomonas putida* KT2440 to investigate the influence of exogenous protein on bacterial physiology. The rationale behind selecting *Pseudomonas putida* KT2440 is, being quorum sensing (QS) negative and lacking machinery for secreting extracellular proteases [19], it can be contended that appearance of phenotypic variation consequent to addition of exogenous protein will be largely independent of QS and nutritional requirement. In this study, we have attempted to evaluate the influence of exogenous protein on growth and metabolism of *P.putida*. Likewise, by considering the fact that protein exerts its influence by virtue of its structure, we have also attempted to investigate capability of different proteins to influence the growth of *P.putida*. Results of our studies underline the ecological significance of extracellular protein mediated growth effects in *P. putida*.

## Results and Discussion

**Figure 1:**
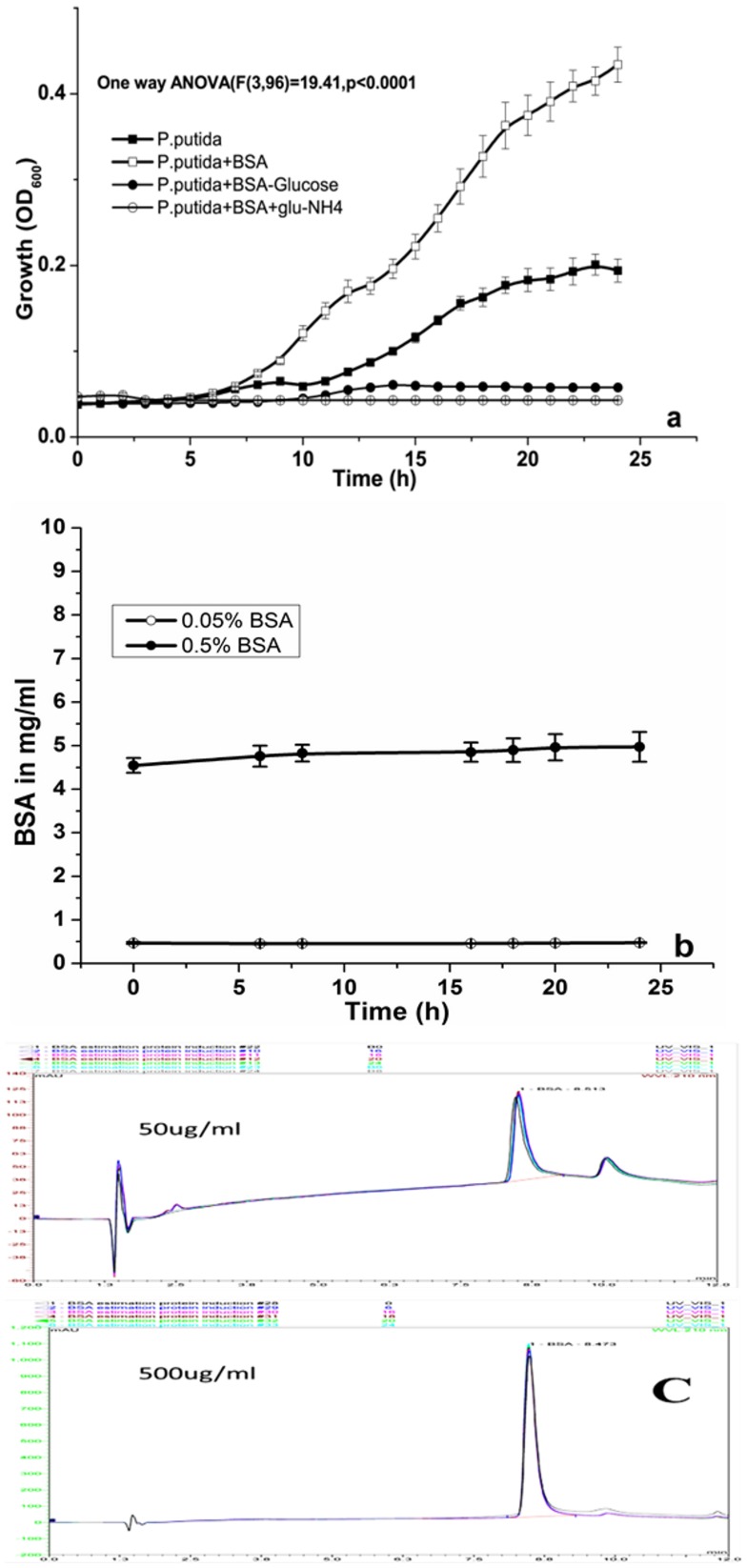
Effect of BSA on growth of *P.putida*. **a**) Effect of BSAaddition (0.5%) on growth of *P.putida* in complete media as well as in media devoid of either carbon source (glucose) or nitrogen source (ammonia). **b**) Residual concentration of BSA in supernatant at different time intervals. Concentration was determined using HPLC (mean±SD). **c**) HPLC chromatogram overlay showing identical peak area of BSA at different time intervals (BSA concentration 0.05% and 0.5%, w/v).

Serum proteins have been shown to influence the growth of pseudomonads such as *P. aeruginosa* [17], [18], [23]. Therefore, we have attempted to investigate the influence of bovine serum albumin (BSA) on the growth of *P.putida*. To determine the influence of BSA on growth, 10^6^ cells of *P.putida* KT2440 were inoculated into 100 ml of Tris media supplemented with glucose and 0.5% BSA (w/v). As shown in figure 1a, there was significant growth enhancement (One way ANOVA: (F(3,96) = 19.41, p<0.0001) in response to addition of BSA, compared to control (maximum growth rate; *P.putida* = 0.11 and *P.putida*+ BSA = 0.024). This observation raises two possibilities: 1) BSA might be acting as carbon or nitrogen source, resulting in enhanced growth or 2) BSA might be acting as inducer of growth, without being used as a carbon or nitrogen source. In order to verify this, two sets of experiments were carried out, where 10^6^ cells of *P.putida* were inoculated intoTris minimal media supplemented with 0.5% or 0.05% of BSA. The media was devoid of either nitrogen source (ammonium chloride) or carbon source (glucose). As shown in figure 1a, there was absolutely no growth in both the cases (i.e. media devoid of nitrogen source or carbon source) compared to the control. Furthermore, as shown in figure 1b, in spite of luxurious growth, protein (BSA) concentration in the medium remained the same after 24 h.An identical peak area in HPLC chromatogram (figure 1c) and absence of lower molecular weight band on SDS-PAGE (figure S1) of various samples taken at different time intervals further confirm that BSAwasnot utilized/degraded by *P.putida* as a source of carbon or nitrogen. This observation is in agreement with the reported inability of *P.putida* KT2440 to utilize BSA,because of lack of secretory proteases. [24], [25].

**Figure 2:**
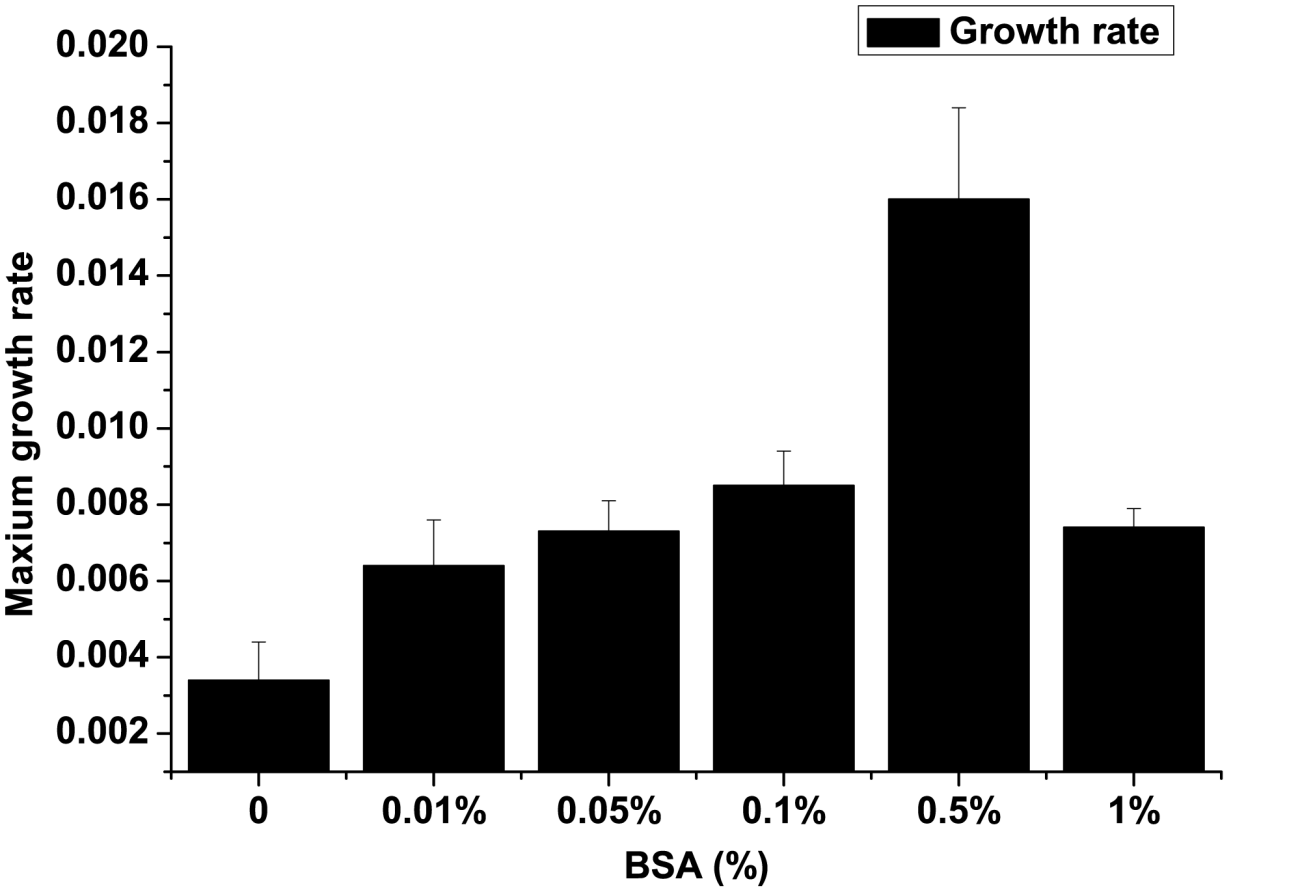
Influence of different concentrations of BSA on growth rate of *P.putida* (mean±SD).

Most of the effecter molecules exert their effect in a concentration-dependent manner and therefore, one may expect similar concentration dependent effect of BSA on the growth of *P.putida*. As shown in figure 2, there was significant enhancement in growth rate in response to increasing concentration of BSA. Significant increase in growth rate was observed up to 0.5% BSA, and thereafter, there was a reduction in growth rate. This observation can be explained in terms concentration dependent effect of signaling molecule, where its effect is significantly reduced or the compound even becomes inhibitory at higher concentration [26]. A classical example is antibiotics which have been shown to have signaling role at low concentrations but become inhibitory at higher concentration [27].

**Figure 3:**
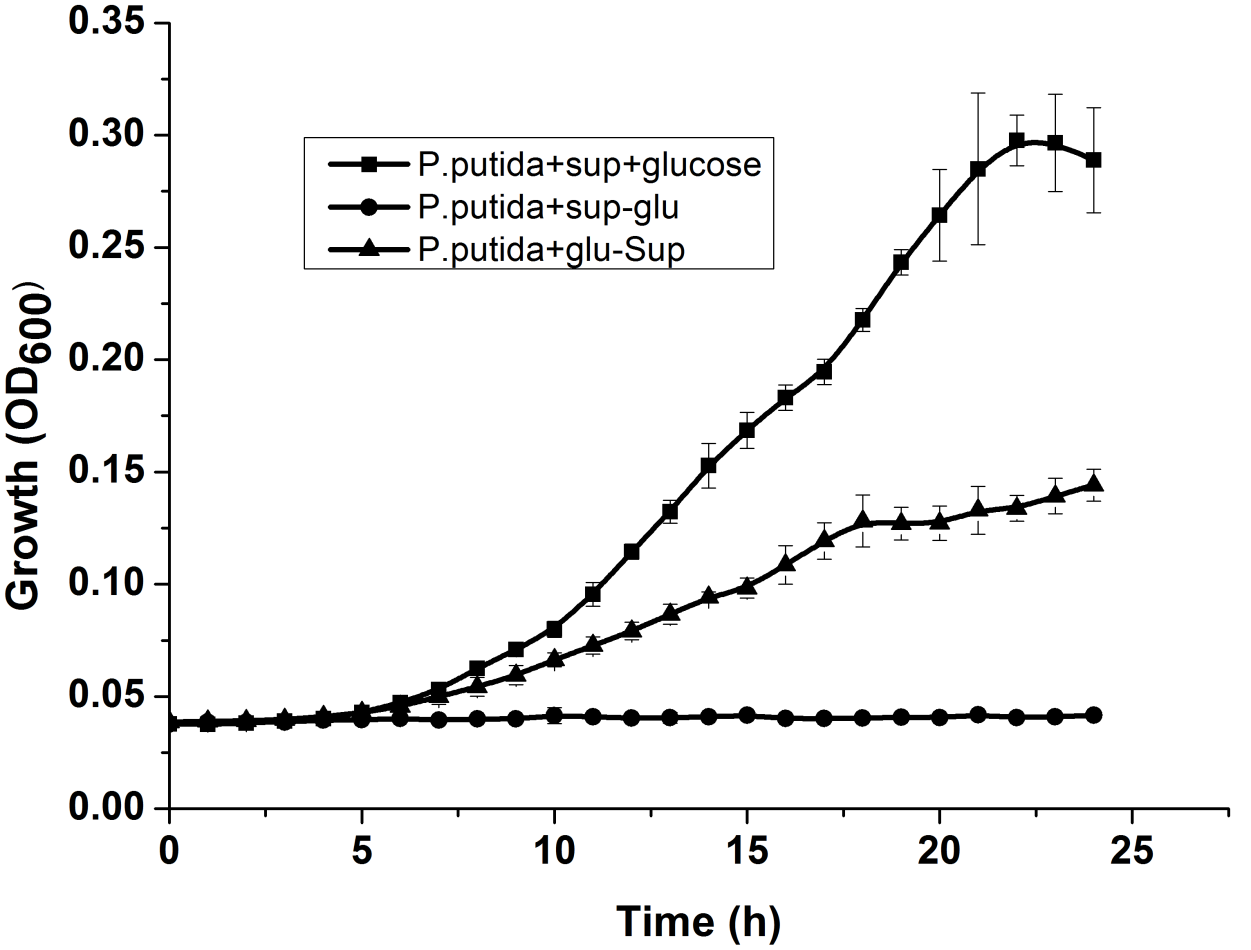
Effect of exogenous proteins (extracted from granular sludge) on growth of *P.putida* (mean±SD).

Presence of serum albumin is restricted to higher organisms only and therefore it was thoughtnecessary to verify growth enhancement in presence of other natural proteins. Previously, *P.putida* has been shown to successfully integrate and proliferate followingbioaugmentation in activated sludge and microbial granules [28]–[30]. Therefore, to evaluate the influence of natural proteins on growth of *P.putda*, we have extracted total proteins from aerobic microbial granules. The extracted proteins were addedat a final concentration of 0.01% intoTris minimal media supplemented with 1% glucose. As shown in figure 3, there was significant growth enhancement (one way ANOVA: F(2,45) 11.36, p<0.001) in *P.putida* inoculated in protein containing Tris-minimal media, compared to growth of *P.putida* in media supplemented with only glucose. The controls were either devoid of carbon source or the inoculum did not show any growth, clearly establishing that the observed growth enhancement was mainly due to the presence of the exogenous proteins.

**Figure 4:**
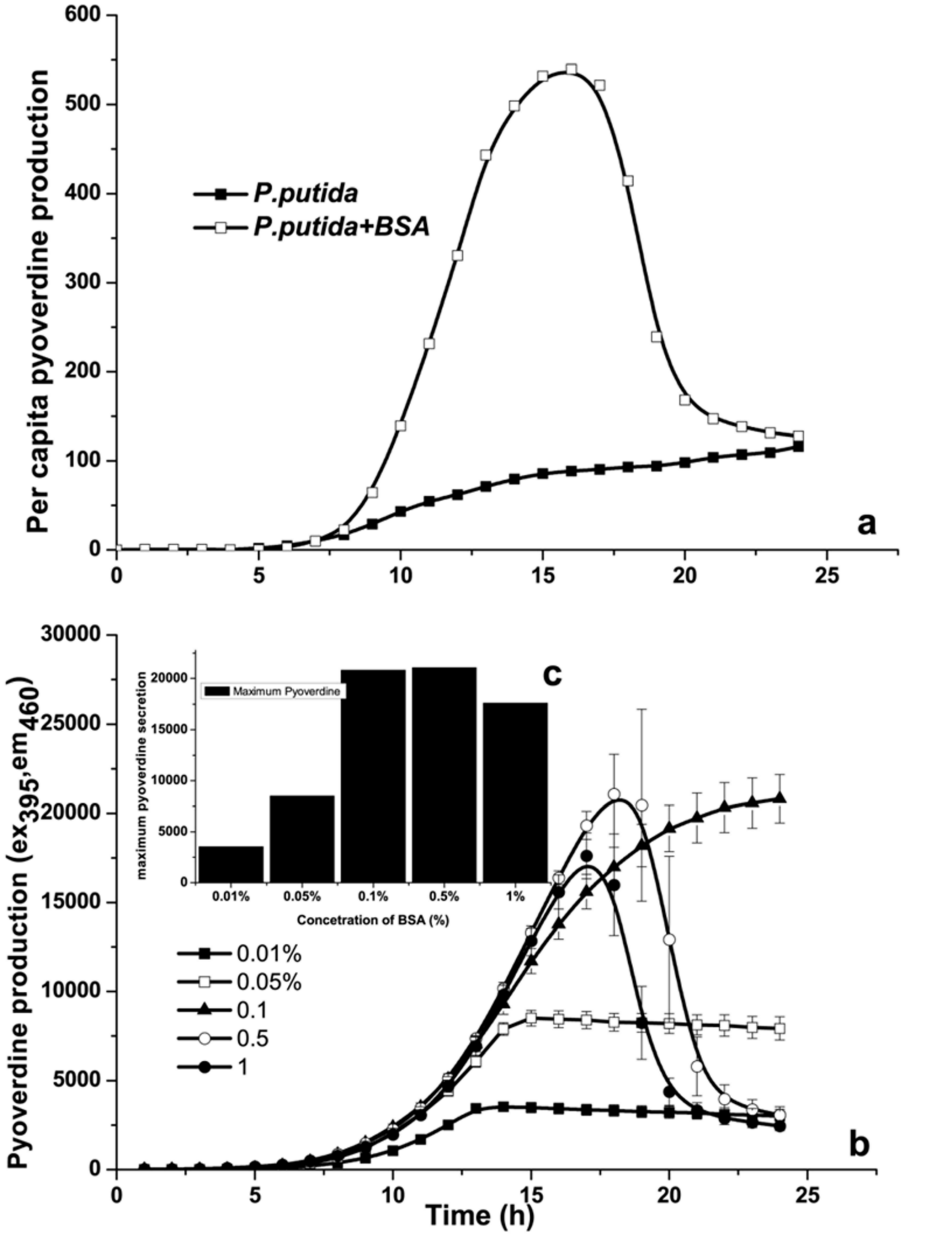
Effect of BSA on siderophore secretion by *P.putida*. **a**) Per capita siderophore secretion by *P.putida* in presence or absence of BSA. **b**) Influence of increasing concentration of BSA on siderophore secretion by *P.putida*. **c**) Maximum siderophore secretion in response to increasing concentration of BSA (mean±SD).

Previously, Kruczek et al [17] have demonstrated significant influence of serum albumin on expression of iron regulatory genes in *P.aeruginosa*. Similarly, Hammond et al [23] have shown that albumin significantly influences motility of *P.aeruginosa*. Interestingly, these two factorswere shown to be closely associated with each other in *P.putida* KT2440, where siderophore secretion was directly correlated with surface motility [31]. Taking lead from these observations, we have investigated the influence of BSA on siderophore secretion by *P.putida*. As shown in figure 4a, there was significant enhancement in per capita siderophore secretion (i.e., siderophore secretion/cell) in response to exogenous addition of BSA, compared to *P.putida* grown without addition of BSA. Furthermore, as shown in figure 4b and c, the increase in siderophore secretion was found to be linearly increasing with increasing concentration of BSA. Iron being one of the most important micronutrients, enhanced siderophore secretion provides additional iron to the cells, resulting in faster growth. These observations have clearly indicated enhanced siderophore secretion as one of the probable reasons behind BSA mediated growth enhancement in *P.putida*.

**Figure 5:**
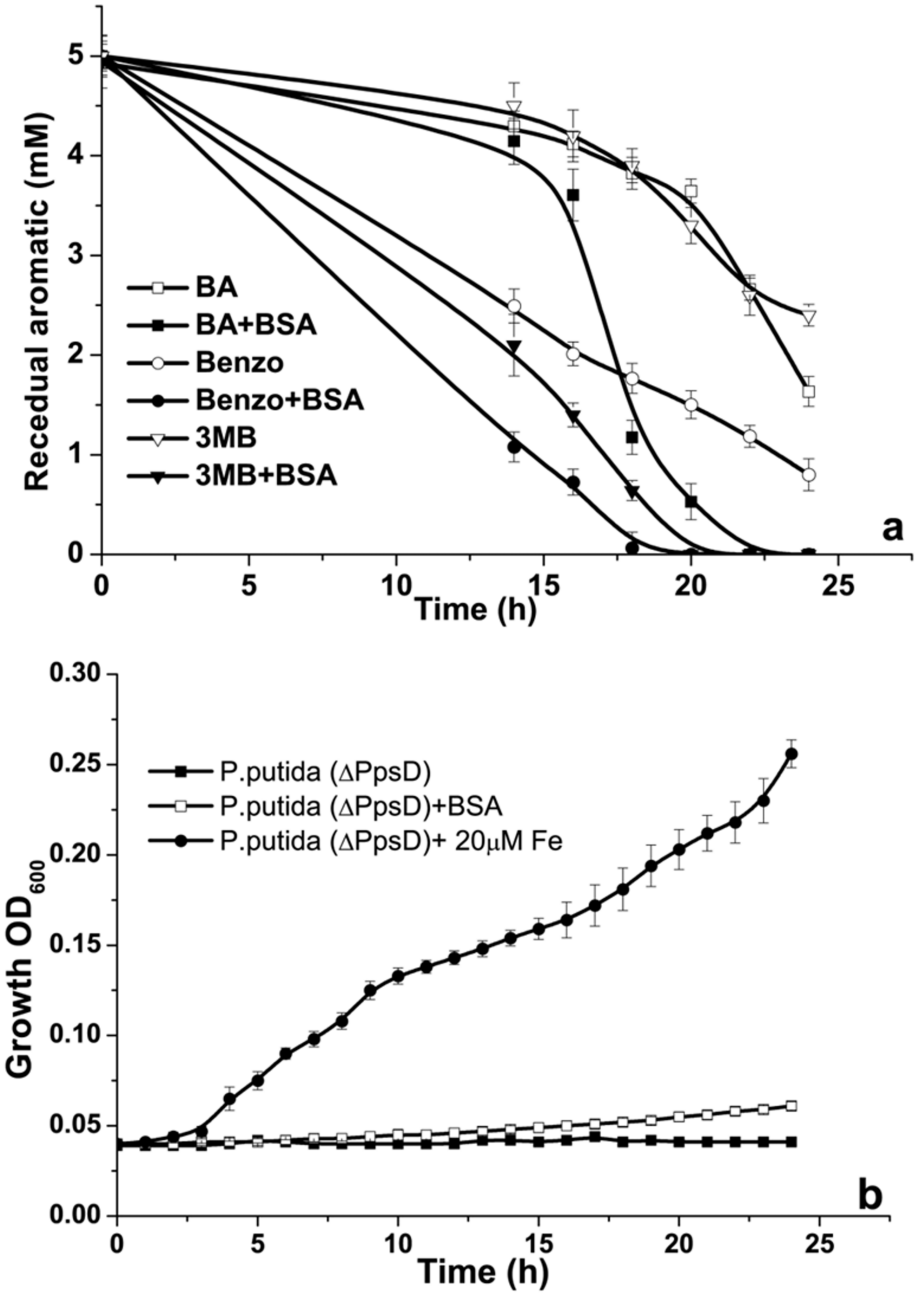
BSA accelerates aromatic substrate utilization by inducing siderophore secretion. **a**) Influence of BSA on various aromatic substrate utilization (**BA**: benzyl Alcohol, **Benzo**; Benzoate, **3MB**; 3-Methyl benzoate). **b**) Influence of exogenously added BSA and Fe^+3^ on growth of mutant (*P.putida*, ΔPpsD) on benzyl alcohol as sole carbon source.

Previously, Gaonkar et al. [32] have reported correlation between siderophore secretion and benzoate (an aromatic substrate) utilization in marine isolates. On a similar note, Dinkla et al. [33] have established strong correlation between iron and toluene utilization by *P.putida*. The fundamental reason behind correlation between iron and aromatic utilization was proposed to be iron dependent oxygenases involved in ring opening pathway. It is logical to expect that enhanced siderophore secretion will have definite impact on aromatic utilization by *P.putida* by providing sufficient iron to the iron dependent oxygenases. As shown in figure 5a, there was significant increase in utilization of benzyl alcohol, benzoate and 3-methyl benzoate (model aromatic hydrocarbon) in response to addition of BSA to the media, compared to *P.putida* grown without BSA addition. Confirmative evidence for BSA mediated enhancement of aromatic utilization was provided using mutant of *P.putida* KT2440 having mutation in the siderophore synthase gene (*P.putida* (ΔPpsD). As shown in figure 5b, growth of *P.putida* (ΔPpsD) on benzyl alcohol was not influenced by addition of BSA, contrary to its wild type, where BSA significantly enhanced the growth. In addition, growth of mutant was significantly enhanced in presence of additional iron provided in the media, in a way similar to the wild type (figure 5b). These observations strongly establish the linkage between exogenous protein induced siderophore secretion and aromatic utilization.

**Figure 6:**
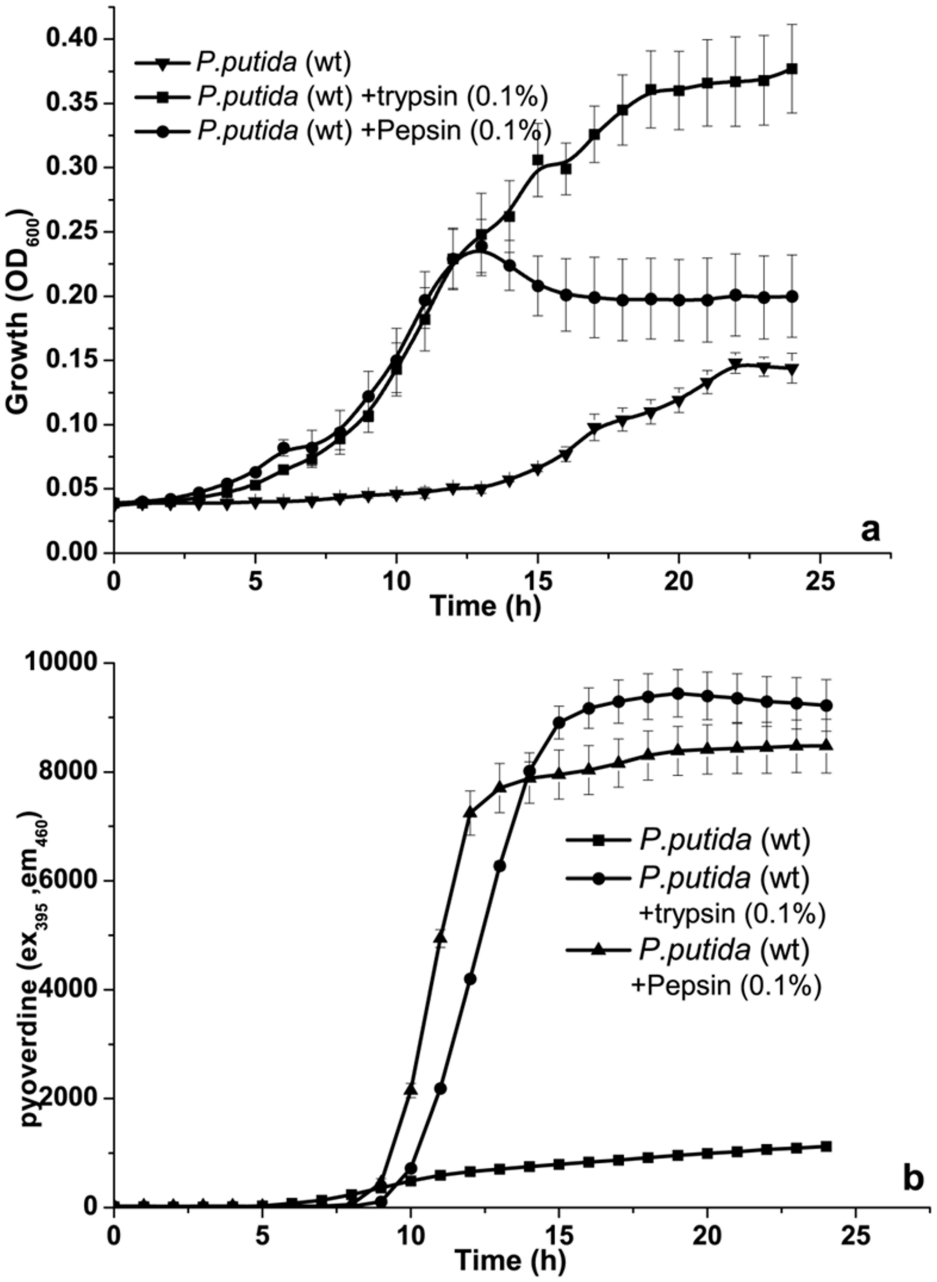
Influence of model proteases on a) growth of *P.putida* and b) Siderophore (pyoverdine) secretion by *P.putida*.

In natural environment, most of the microbial secretory proteins have enzymatic role and are of digestive nature [34]. Similarly, good amount of plant secretory proteins have also been identified as enzymes [35], [36]. These secretory enzymes have been shown to play a key role in pathogenesis, nutrient uptake, biocontrol and colonization [37]–[39]. In addition, secretory proteases are known to play significant role in competitive interaction among microbes [40]. Moreover, there are many reports on the role of plant secretory enzymes on selective proliferation of beneficial microbes [5]. These observations point to the central role played by enzymes in community dynamics in natural environment. Therefore, it would be interesting to know how *P.putida* responds to these enzymes (proteins). We investigated the influence of two model protease enzymes, namely trypsin and pepsin, on the growth of *P.putida*. As shown in figure 6a and 6b, there was significant growth enhancement concomitant with increase in siderophore secretion. Growth enhancement in response to secretory enzymes will have positive impact on competitive survival of *P.putida* in presence of other occupants of its rhizospheric environment, which employ secretory proteases to utilize proteinaceous plant exudates and to inhibit colonization of other microbes. The present observation also points to the non-specific nature of protein mediated growth enhancement in *P.putida*. At this juncture we are not clear on the overall pathway of protein induced growth enhancement in *P.putida*; this needs to be investigated in detail. Nevertheless, our observation opens up a new avenue in the area of protein mediated signaling in bacteria.

Studies pertaining to microbial communication remain dominated with small molecules like AHL, AI, volatile fatty acids etc. However, there are many emerging reports that have indicated the role played by complex molecules in communication between microbes and microbes and their hosts. Recently, Beauregard et al. [41] have reported sensing of plant-derived polysaccharide by *Bacillus subtilis*, which influences its biofilm formation capability. On a similar note, Antoniuk and Evseeva [42] showed that lectin could act as a communication molecule between plant and microbes. Gallio et al. [43] elucidated the role played by a conserved rhomboid protein in communication between bacteria and higher organisms. Plants secrete a variety of proteins in the form of root exudates, which have been shown to significantly influence the microbial community structure in rhizospheric environment [7], [10], [44]. These observations were corroborated by many studies employing meta-proteomic approach, where proteins were shown to be influencing microbial dynamics of a given ecosystem [45]–[47]. Our observation supports emerging evidence of proteins playing a crucial role in determining microbial community structure. Specifically, we have shown that proteins influence the growth of *P.putida*, in spite of not being used as a source of carbon or energy.

Ecological significance of exogenous protein mediated growth enhancement in *P.putida* can be explained in terms of the central role played by iron in competitive root colonization and persistence of *P.putida* in rhizospheric environment [48], [49]. Consequently, factors influencing iron availability to *P.putida* will have profound impact on its survival and proliferation. Siderophore being the principle molecule responsible for iron uptake, one can expect positive impact of enhanced siderophore secretion on competitive fitness of *P.putida*. Proteins, as mentioned earlier, are among the major constituents in root exudates and are invariably present in all microorganisms. Therefore, we hypothesize its role as that of a chemical cue, facilitating the survival *P.putida* in the highly competitive rhizospheric environment. This presumption becomes more relevant when one considers the fact that *P.putida* can secrete only a single type of siderophore (pyoverdine) and has only limited capability to utilize heterologous siderophores [31]. Therefore, exogenous protein mediated enhanced siderophore secretion will provide an opportunity to *P.putida* to achieve an edge over its competitors to accelerate iron assimilation. Enhanced siderophore secretion will facilitate rapid utilization of aromatic compounds normally present in root exudates [50]. Interestingly, secretion of these aromatic hydrocarbons by plants has been shown to become pronounced under iron starvation conditions [51]–[53]. Consequently, exogenous protein induced siderophore secretion will have two advantages in terms of better assimilation of iron and substrates (aromatic hydrocarbon), which in turn provides the bacterium with fitness advantages over its competitors.

## Materials and Methods

### Strain and growth conditions

All the experiments were carried out using *Pseudomonas putida* KT2440 (*P.putida*), chromosomally marked with DsRed and harboring *gfp*mut3b-modified Kan^R^ plasmid pWWO [20]. Experiments pertaining to establishing siderophore as key molecule involving in exogenous protein mediated growth enhancement was carried out using mutant *P.putida* KT2440 (ΔPpsD) having mutation in siderophore synthetase gene. The mutant was electroporated with plasmid pWWO isolated from wild type. Both the strains were maintained on Luria agar plate and grown overnight in Luria broth before experimentation. Experiments were carried out in 250 ml Erlenmeyer flasks containing Tris-minimal media (Tris, 6.05; Sodium chloride, 4.67; Potassium chloride, 1.5; Ammonium chloride, 1.06; Sodium sulphates, 0.42; Magnesium chloride, 0.233, Calcium chloride, 0.03; Sodium dihydrogen phosphate (dehydrate), 0.004 (All chemicals grams per litre of H_2_0). A stock solution of trace elements was prepared that contained the following (in mg/l): Zinc sulphate (heptahydrate), 143.77; Magnesium chloride (Tetrahydrate), 98.96; Boric acid, 61.83; Cobalt chloride, 190.34; Copper chloride, 17.05; Nickel chloride, 23.77; and sodium molybdate, 26.29. From the trace elements solution 100 µl/litre was added to the above Tris solution) supplemented with either glucose (1%) or 5 mM of aromatic hydrocarbon as sole source of carbon and energy. All the other chemicals were of AR grade, purchased from Merck, Germany.

### Protein induction experiments

For protein induction experiments, different concentration of Bovine Serum Albumin fraction V (BSA) was added into the Tris minimal media containing glucose or aromatic hydrocarbon as sole source of carbon. Similarly, for experiments involving proteases, 0.1% trypsin or pepsin was added to the media in place of BSA and rest of the experimentation was carried out identically. For experiment involving natural proteins, total protein was isolated from aerobic granular biomass obtained from laboratory scale bioreactor (granular biomass or granular sludge is a multispecies microbial consortium compactly packed in the form of spherical granules. It has been cultivated in sequencing bioreactor using acetate as sole source of carbon) as follows: 100 ml of effluent was collected and centrifuged at 8000 rpm, for 5 min. After discarding supernatant, the biomass was washed twice with Tris-minimal media and finally re-suspended in 10 ml of Tris minimal media. Total protein was isolated after sonication (5×20 sec, 32% intensity). After sonication, the biomass was centrifuged at 10000 rpm for 5 min to remove cell debris. Proteins present in the supernatant was further precipitated by TCA (20% v/v) and subsequently TCA was removed by multiple washes with cold acetone. Finally, protein was dissolved in 1 ml of water and concentration was determined using BCA protein estimation kit from Pierce. For induction experiments, 10 ml of overnight grown culture of *P.putida* (grown in LB) was taken and centrifuged at 7000 rpm for 3 min. After centrifugation, supernatant was discarded and cell pellet was washed 3 times with 10 ml of sterile 0.85% NaCl, w/v. Finally, the cells were re suspended in 10 ml of sterile normal saline, from which 10^6^cells of *P.putida* were inoculated into 250 ml flask containing 100 ml Tris minimal media. For growth, substrate utilization and siderophore secretion, samples were drawn at different time interval and analyzed accordingly.

### Determination of growth curve and siderophore (pyoverdine) production

For determination of growth and siderophore secretion under influence of exogenous protein, we have used automated multimode reader (Biotek Synergy H5, Germany), which has the capability to simultaneouslymonitor the OD and fluorescence in 96 well microtitre plates. *P.putida* cells (10 µl of 10^6^ cells/ml were inoculated into 150 µl of Tris media for all the experiments. The microtitre plates were incubated at 30°C with intermittent shaking. Growth and pyoverdine production were monitored by measuring optical density (600 nm) and fluorescence (395_ex_, 460_em_), respectively, at hourly intervals using the kinetic mode of the reader. Bacterial density in cfu/ml was calculating by plotting OD vscfu/ml calibration plot. Similarly, secreted pyoverdinewas confirmed and quantified using universal Chrome azurol S (CAS) assay as described [21].

### HPLC analysis

Residual benzyl alcohol, benzoate and methyl benzoate were estimated using Dionex Ultimate 3000 HPLC system fitted with an auto-sampler. The samples were centrifuged at 10,000 rpm for 2 min and 1 ml of supernatant was introduced into the auto-sampler vials. The HPLC operating parameters were: mobile phase - acetonitrile: water (20∶80)+0.1% (v/v) phosphoric acid, flow rate 1 ml/min, column - BondapackC_18_ RP; and detection at 254 nm using UV-Visible detector. Similarly, residual BSA in supernatant was estimated using two methods. 1) BCA protein estimation kit (Pierce) against BSA standard and 2) by HPLC with following operational parameters: mobile phase – 0.1% TFA in water and 0.1% TFA in acetonitrile (gradient 95∶5 to 35∶65 in 10 min), flow rate 1 ml/min, column - BondapackC_18_ RP; and detection at 210 nm using UV-Visible detector.

### Calculation and statistics

Growth rate was calculated using an online tool (http://modelling.combase.cc/DMFit.aspx).

1. Per capita siderophore secretion was calculated using the following formula:
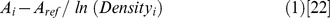
Where,
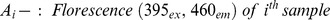


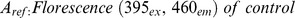


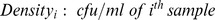

## Conclusions

- There was significant enhancement in growth of *P.putida* under the influence of exogenous protein even when itdidnot act as a carbon or nitrogen source.
- The growth enhancement was significantly influenced by the concentration of exogenous protein present in culture medium (dose dependence)
- Protein induced growth enhancement was mediated via increase in siderophore secretion, with resultant increase in iron assimilation and faster growth.
- Enhanced siderophore secretion mediated by exogenous proteins leads to increase in aromatic (benzyl alcohol, benzoate, 3-methyl benzoate) utilization. This increase in aromatic utilization is presumed to be due to augmented iron supply to the iron dependent oxygenases responsible for ring opening pathway.
- Protein-induced siderophore secretion and consequent growth enhancement were non- specific in nature,with different types of proteins exerting comparable effects.
- It is hypothesized that exogenous proteins act as chemical cue for *P.putida*, that perceive them as indicator of the presence of potential competitors in the environment. Such chemicalwarning of the presence of competitors in rhizospheric environments would allow *P.putida* to accelerate its iron assimilation and thereby growth, resulting in better survival.

## Supporting Information

Figure S1 **Image of SDS-PAGE showing intact BSA at different incubation time period.**
(TIF)
